# Unveiling Lichen’s Hidden Arsenal Against Multidrug Resistance: A Systematic Review of Their Essential Oils, Volatile Compounds and Extracts with Antimicrobial Applications

**DOI:** 10.3390/microorganisms14040924

**Published:** 2026-04-20

**Authors:** Yasser Essadki, Soukaina El Amrani Zerrifi, Maria de Fátima Carvalho, Lillian Barros, Vitor Vasconcelos, Alexandre Campos, Fatima El Khalloufi, Brahim Oudra, Rosário Martins

**Affiliations:** 1 Water Sciences, Microbial Biotechnologies and Sustainability of Natural Resources Laboratory (Aquabiotech), Faculty of Sciences Semlalia of Marrakech, Cadi Ayyad University (UCA), Av. Prince My Abdellah, P.O. Box 2390, Marrakech 40000, Morocco; yasser.essadki@ced.uca.ma (Y.E.); s.elamrani@sante.gov.ma (S.E.A.Z.); oudra@uca.ac.ma (B.O.); 2 Higher Institute of Nurses Professions and Health Techniques of Dakhla, Dakhla 73000, Morocco; 3 Interdisciplinary Centre of Marine and Environmental Research (CIIMAR), Terminal de Cruzeiros do Porto de Leixões, Av. General Norton de Matos, s/n, 4450-208 Porto, Portugal; mcarvalho@ciimar.up.pt (M.d.F.C.); vmvascon@fc.up.pt (V.V.); acampos@ciimar.up.pt (A.C.); 4 ICBAS—School of Medicine and Biomedical Sciences, University of Porto, Rua de Jorge Viterbo Ferreira 228, 4050-313 Porto, Portugal; 5 CIMO, LA SusTEC, Instituto Politécnico de Bragança, Campus de Santa Apolónia, 5300-253 Bragança, Portugal; lillian@ipb.pt; 6 Department of Biology, Faculty of Sciences, University of Porto, Rua do Campo Alegre, 4169-007 Porto, Portugal; 7 Natural Resources Engineering and Environmental Impacts Team, Multidisciplinary Research and Innovation Laboratory, Polydisciplinary Faculty of Khouribga, Sultan Moulay Slimane University of Beni Mellal, P.O. Box 145, Khouribga 25000, Morocco; f.elkhalloufi@usms.ma; 8 ESS, Escola Superior de Saúde, Polytechnic of Porto, Rua Dr. António Bernardino de Almeida 400, 4200-072 Porto, Portugal

**Keywords:** lichens, essential oils, volatile organic compounds (VOCs), systematic review, PRISMA 2020, multidrug resistance, antimicrobial activity, secondary metabolites

## Abstract

The increase in multidrug resistance in microorganisms and the rise of emergent infectious diseases worldwide is a threat to human and animal health. Therefore, research on new molecules with antibiotic potential is a priority. Lichens have a unique secondary metabolism with relatively untapped potential, yet their essential oils (EOs) and volatile organic compounds (VOCs) remain a relatively untapped resource. This systematic review was conducted following PRISMA 2020 guidelines, with a comprehensive search performed in the Web of Science database for studies published up to 2023. From 254 identified records, six studies involving nine lichen species (*Evernia prunastri*, *Evernia divaricata*, *Cladonia rangiformis*, *Cladonia furcata*, *Parmotrema perlatum*, *Lichina pygmaea*, *Parmelia perlata*, *Hypogymnia physodes*, and *Parmelia sulcata*) met the eligibility criteria. The synthesized data show that these volatile fractions possess significant antimicrobial potential, with minimum inhibitory concentrations (MICs) generally lower than 1 mg/mL. Major bioactive constituents identified include atraric acid, orsellinates, and various sesquiterpenes. While the current evidence highlights a strong potential of lichen volatiles against pathogens, research is limited to a small fraction of known species. This review identifies a critical gap in testing these compounds directly against MDR clinical isolates and suggests that future research should focus on high-biomass species and the heterologous expression of lichen biosynthetic genes to develop sustainable antimicrobial applications.

## 1. Introduction

Multidrug-resistant microorganisms and emergent infectious diseases are a public health threat worldwide [1,2]. According to Murray et al. [3], in 2019, 4.95 million deaths globally were attributed to drug-resistant infections. Among the critical steps in mitigating the impact of antimicrobial resistance is the constant investment in developing new antimicrobial compounds [3]. Nature has been found to be a viable source of antibiotics [4]. Molecules such as abyssomicins produced by *Verrucosispora* sp., for instance, have shown interesting potential as an antitubercular pro-drug [5], while capsaicin and dihydrocapsaicin isolated from *Capsicum annum* demonstrated potential against Methicillin-resistant *Staphylococcus aureus* (MRSA) with efflux pumps and also against R-plasmid conjugal transfer [6]. In this context, lichens are considered a relatively untapped resource with a unique secondary metabolism [7]. A range of compounds, generally obtained by organic solvent extraction followed by fractionation have already been assessed for their antimicrobial potential [8]. For instance, usnic acid, a compound isolated from *Usnea*, *Cladonia*, *Lecanora*, *Ramalina* and *Evernia* species, has shown activity against a wide range of microorganisms [9]; atranorin and fumarprotocetraric acid, isolated from *Cladonia foliacea*, have also demonstrated antimicrobial potential [10]; and other compounds such as barbatic acid from *Cladonia borealis* [11], diffractaic acid from *Usnea blepharea* [12], divaricatic acid from *Evernia mesomorpha* [13], evernic acid from *Evernia prunastri* [14] and lecanoric acid from *Melanelia subaurifera* and *Melanelia fuliginosa* [15] have also shown antimicrobial potential [8].

While the antimicrobial potential of lichens has been the subject of several comprehensive reviews, the vast majority of the existing literature focuses on “classical” secondary metabolites derived from organic solvent extracts [16,17]. The volatile fraction, consisting of essential oils and low-molecular-weight VOCs, remains largely untapped and poorly synthesized. This study represents the first systematic review, conducted under the PRISMA 2020 framework, to focus exclusively on these volatile components. Beyond merely cataloging antimicrobial activity, this review provides a novel comparative analysis between volatile fractions and traditional extracts for the same species and establishes a mechanistic framework for their application against multidrug-resistant (MDR) pathogens, thereby addressing a critical gap in current lichenological and pharmacological research. In this review, we highlight the gap in the literature regarding this specific type of compound from lichens. We also propose an explanation as to why the gap exists and what strategies should be used to optimize future work on this aspect.

## 2. Materials and Methods

### 2.1. Search Strategy and Information Sources

This systematic review was conducted in accordance with the PRISMA 2020 (Preferred Reporting Items for Systematic Reviews and Meta-Analyses) guidelines. A comprehensive literature search was performed in the Web of Science (WoS) (all databases). The search was conducted in March 2026, covering all records indexed from the database’s inception until December 2023. The primary search string utilized was: lichen* AND (“essential oil*” OR “volatile compound*” OR “volatile organic compound*”). To ensure the capture of all relevant literature, the database search was supplemented by citation searching and handsearching of retrieved reports, which identified one additional relevant study by Maqbul et al. [18] that did not contain the keyword “lichen” in its metadata.

### 2.2. Inclusion and Exclusion Criteria

To fulfill the objectives of this review, studies were selected based on the following inclusion criteria:

1. Primary research articles focusing on lichen-forming fungi.
2. Extraction of volatile fractions specifically via steam distillation, hydrodistillation, or Clevenger-type apparatus, following the definitions by Bicchi and Joulain [19].
3. Chemical characterization of the volatile profile using gas chromatography–mass spectrometry (GC-MS) or similar methods.

The exclusion criteria were: (i) studies focusing exclusively on traditional organic solvent extracts (e.g., acetone, methanol) without a dedicated volatile analysis; and (ii) review articles, conference proceedings, or book chapters.

### 2.3. Study Selection and Screening

**Figure 1:**
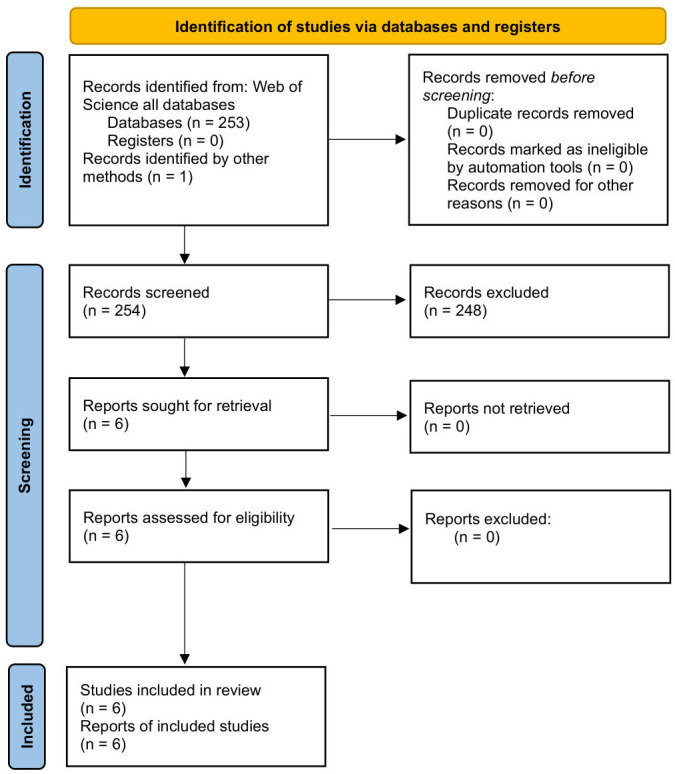
PRISMA 2020 flow diagram for the systematic review of lichen essential oils and volatile compounds.

The selection process was summarized using a PRISMA flow diagram (Figure 1). Initially, two reviewers (Y.E. and S.E.A.Z.) independently screened the titles and abstracts of the *n* = 254 identified records. Discrepancies were resolved through consensus or consultation with a third reviewer (B.O). After the removal of irrelevant records (*n* = 248), the full-text versions of the remaining six articles were retrieved and assessed for eligibility. All six reports met the criteria and were included in the qualitative synthesis.

### 2.4. Data Extraction and Synthesis

Data were extracted from the included studies regarding: lichen species, geographical origin, extraction yield, major chemical constituents (with Kovats retention indices), and antimicrobial parameters against target pathogens. For the six species where antimicrobial data were available, an additional targeted search was performed using the species name and the terms “antimicrobial,” “antibacterial,” or “antifungal” to retrieve data on their respective organic solvent extracts for comparative analysis. This allowed for a qualitative synthesis comparing the potency of volatile fractions against established secondary metabolites. We noted that no data were found for *Lichina pygmaea* (Lightf.) C. Agardh organic solvent extracts.

### 2.5. Risk of Bias and Quality Assessment

The quality of the included studies was assessed based on the standardization of the antimicrobial assays (e.g., use of ATCC reference strains) and the rigor of the chemical identification (e.g., confirmation of constituents via both mass spectra and retention indices). As this is a qualitative systematic review of laboratory data, a formal meta-analysis was not performed.

## 3. Lichen General Characteristics

Lichens are the macroscopic representation of lichen-forming fungi in their symbiotic state with a photosynthetic microorganism [20]. They are generally composed of an ascomycete as an “exhabitant” heterotrophic organism, referred to as a mycobiont in the context of the symbiosis, and a green microalgae as an “inhabitant” autotrophic microorganism, referred to as a photobiont in the context of the symbiosis [20,21,22,23]. The mycobiont presumably provides a favorable environment for the photobiont to develop and helps to aggregate essential minerals and oligo-elements from airborne dust by a mechanism that remains to be fully described [24,25], whereas the photobiont produces photosynthates useful to the mycobiont.

A metabolic coupling loop has indeed been uncovered between the mycobiont and the photobiont. The mycobiont, consuming O_2_ and photosynthates produced by the photobiont, releases CO_2_ that is in turn used to produce photosynthates by the photobiont under light conditions [26]. With regard to these two essential elements of the organism, a large number of exceptions to the general rule are known and new ones keep being discovered. Currently, the photobiont is a Chlorophyceae in 90% of cases and a Cyanophyceae in 10% of known lichens [21,23]. Similarly, the mycobiont in most lichens is an ascomycete, although in some cases it is a basidiomycete [27]. However, “composite/tripartite” lichens exist too, comprising a mycobiont associated with a Chlorophyceae and with pockets of Cyanophyceae called “cephalodia” [21,23]. Lichen symbiosis also involves an associated microbiome of lichen-associated fungi [25], lichenicolous fungi [28], bacteria (alphaproteobacteria and actinobacteria, among others) [23], viruses [29,30,31], and protozoa [32].

A microecosystem view of these organisms has been proposed, supported by the extreme difficulty of resynthesizing macroscopic lichen in the laboratory under axenic conditions [33,34,35,36,37]. This showcases the actual lack of knowledge on the morphogenesis mechanisms in this group [21].

Lichens are commonly described based on their thallus form. Honegger [38] described the main classes of lichen thalli (Table 1).

For instance, gelatinous lichens, such as *Collema* or *Lichina*, have a characteristic thallus that swells in the presence of water and shrinks in dry conditions. The crustose lichens are the most widespread and display photobionts in high proportions in comparison with the mycobiont, and scattered on the thallus, they are devoid of stratification and peripheral cortices. The foliose and fruticose lichens have the most complex thalli, with a stratified architecture, an inner and outer cortex, and a dorsi-ventral differentiation, forming a leaf-like structure for the foliose lichens and a plant-like structure for the fruticose lichens [38,39].

The taxonomical consensus regarding the naming of lichens (macroscopic organisms) is the use of the name of the mycobiont (lichen-forming fungi) as a descriptor for the whole organism, without citing the photobiont, which can be referred to using the classical nomenclature [25]. Furthermore, around 20,000 species are recognized worldwide [27]. New species are discovered at a high rate using molecular methods in biodiversity hotspots such as Colombia and Brazil [40,41].

Lichens are extremely resilient organisms, with a very flexible and condition-dependent metabolism [42]. Their metabolism can be slowed to the point of nearly stopping in the absence of water, but as soon as it is present, it restarts and runs at full speed [38,43]. They are also very resistant to UV light [42,44,45]. These characteristics explain the fact that they occupy a very wide ecological niche, ranging from deserts to forests, high-altitude, low-temperature areas, and volcanic islands [25,38,44,46]. They can be understood as pioneer organisms that colonize hostile media and partake in rendering them hospitable for other organisms like vascular plants by eroding the bedrock and generating the first layers of soil in newly formed volcanic islands, for example [47,48,49,50,51].

One noticeable characteristic of lichens is their relatively slow growth rate, which ranges from less than 1 mm to a few millimeters per year [38]. Nevertheless, lichen growth rate is dependent on environmental conditions, and the same species can grow at various rates [52]. The genii *Lobaria* and *Ramalina* have representatives with the highest growth rates. A growth rate of around 43 cm per year has been recorded for *Ramalina menziesii* [38].

Lichens have been used by researchers as bio-indicators of pollution by exploiting their mineral- and oligo-element-accumulating potential, which extends to pollutants, radionuclides, and heavy metals [38]. Therefore, some lichens are tolerant of pollution, or rather thrive in it, while others tend to disappear in polluted areas and be present in areas with better air quality [25,38].

Traditional uses of lichens by human populations are relatively anecdotal compared to those of other organisms such as vascular plants, probably because of the relatively low biomass availability and the difficulties associated with their harvesting, but also because of the slow regeneration rate of these organisms [53]. Various cultures use lichens as food, medicine, dyes, cataplasm, spice, and fodder [53,54]. In the polar region, lichens such as *Cladonia* sp. are consumed by the herds of *Rangifer tarandus* as forage during the winter period [55,56,57]. However, the fragrance and dye industries are the only ones to use lichens on an industrial scale [54,58,59].

## 4. Secondary Metabolites from Lichens

### 4.1. Overview of Some Lichenic Compounds

**Figure 2:**
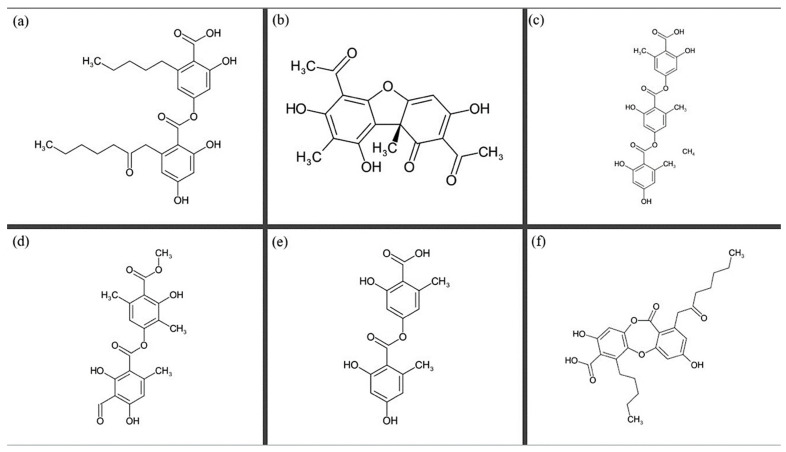
The molecular structure of some secondary metabolites from lichens. (**a**) Olivetoric acid. (**b**) Usnic acid. (**c**) Gyrophoric acid. (**d**) Atranorin. (**e**) Lecanoric acid. (**f**) Physodic acid.

Lichens produce a wide variety of secondary metabolites, somewhat different from those produced by vascular plants [60]. Approximately one thousand molecules have been identified in lichens [8,60]. Because of the microecosystem nature of lichens, the metabolites, probably produced by the mycobiont, can undergo transformations mediated by the microorganisms associated with the lichen [25]. They tend to be deposited as crystals in the cortex outer layer, as shown by numerous electron microscope images [61,62,63,64]. The biosynthetic pathways of these secondary metabolites mainly pass through the shikimic acid path, the mevalonic acid path, or the polyketide path, or are derived from photobiont photosynthetic products [60,65]. The major classes of secondary metabolites produced by lichens are depsides, depsidones, depsones, dibenzofurans, xanthones, anthraquinones, and chromones [60,65]. Moreover, atranorin, usnic acid, gyrophoric acid, lecanoric acid, olivetoric acid, and physodic acid are among the most widely studied secondary metabolites of lichen origin for their biological activities, and several reviews have already been published about them [8,66]. This section consists of an overview of the most commonly studied secondary metabolites from lichens, their structure, biosynthetic pathways, antimicrobial potential, other bioactive potential and toxicity (Figure 2, Table 2).

Several organic extracts of lichens have been studied. Acetone, methanol, ethyl acetate and hexane are the main solvents used in lichen compound extraction for the screening of their biological activities. The most common protocol of extraction involves biomass grinding, and then maceration in the solvent for some time with agitation (generally 24 to 48 h), or successive macerations with renewed solvents, before filtering, mixing the solvent batches together and evaporating the solvent in a Rotavapor to retain the extract. Soxhlet extractions using the same types of solvents at 50 °C for 12 h is also relatively common [65,82,83,84,85].

Most of the extracts contain the main lichenic compounds in different proportions, as shown by HPLC analysis in numerous studies [65,86]. Nevertheless, the acetonic extracts seem to be the most bioactive overall against the microorganisms evaluated [86,87].

Acetone is one of the most used solvents for extracting lichen secondary metabolites because it can solvate compounds with a wide range of polarities; it was also historically used in the microscopic identification of lichenic compounds using the microcrystal test [65]. Komaty et al. [87] conducted an optimization experiment, trying to find the most efficient pulverization method and the best solvent for extracting secondary metabolites from lichens, guiding their work using scanning electron microscopy. Their findings pointed towards grinding with an electrical grinder and using acetone for extraction as the most efficient way to extract microscopically visible crystal deposits of secondary metabolites from the thallus.

Some mentions are made in the literature of aqueous extracts of various lichen species. Unfortunately, very few chemical characterizations of these types of extracts were made. In fact, the antimicrobial activity of aqueous extracts is quite rare, even nonexistent, in the literature investigated. The main hypothesis as to why these types of extracts are ineffective is that the secondary metabolites from lichens are insoluble in water and therefore are not extracted, or only in extremely low yields, by these methods. Another explanation for this lack of activity might be the use of ineffective extraction procedures. We, therefore, suggest that after extracting the lichen biomass with water, and filtering the aqueous extract with 0.2 µm sterile syringe filters to get rid of contaminants, freeze-drying them in order to be able to assess the yield of extraction and meticulously control the concentrations used in the tests.

The characterization of the composition of “classical” (acetone, methanol, and hexane, among others) extracts of lichens in articles discussing their antimicrobial potential tends to be done using HPLC methods and standards of major lichen compounds such as atranorin. Another means of qualitative characterization of the extracts is the determination of the total phenolic compounds and total flavonoid content. These methods are quite solid, but the main drawback in our opinion is that minor compounds of the extracts may be responsible for the activity observed, and therefore, a more thorough investigation and fractionation of the composition of the extracts is needed to assess the real potential of the different compounds contained in them.

### 4.2. Antimicrobial Activity of Volatile Compounds and Essential Oils from Lichens

Volatile organic compounds (VOCs) are defined by the European Union as any compound with a vapor pressure of 0.01 kPa or more at a temperature of 293.15 °K or with a corresponding volatility under the particular conditions of use [88]. This definition includes volatile solvents and chemical derivatives of industrial origin [88]. These compounds are generally composed of carbon and hydrogen, with substitution of some of the hydrogens on the hydrocarbon chain by halogens, oxygen, sulfur, silicon, nitrogen, and phosphorous, except for carbon oxides, and inorganic carbonates and bicarbonates [88].

Alternative definitions exist, such as compounds that show more than 95% by-weight evaporation after 6 months under ambient conditions [89]. These compounds, especially those of anthropogenic origin, are considered potentially harmful to the environment as they are capable of evaporating, disseminating, and transforming into other compounds [88]. Nevertheless, VOCs are also produced by a wide array of organisms, including plants, seaweeds, and microorganisms [90]. Biogenic VOCs of vegetal origin seem to play a role in the ecosystem–atmosphere regulation loop [91,92].

Essential oils are derived from vegetable raw materials, and are obtained through various methods such as water or steam distillation, extraction from the epicarp of *Citrus* spp. fruits via a mechanical process, or dry distillation. After extraction, the essential oil is separated from the aqueous phase by physical means [19]. The components of essential oils are volatile because they have boiling points low enough for distillation, particularly atmospheric-pressure steam distillation. Thus, the components have molecular weights below 300 Daltons (with molecular mass relative to hydrogen being 1) and tend to be fairly hydrophobic [93]. Essential oils contain a mixture of VOCs, either directly biosynthesized by the organism from which they are extracted, or indirectly, by chemical transformation of precursor compounds produced by the organism [94]. They are generally composed of monoterpenes, sesquiterpenes, and diterpenes. Additionally, they often contain phenylpropanoids, fatty acids and their esters, or their decomposition products as volatile components [95].

The distinction should be made, as pointed out by Bicchi and Joulain [19], between essential oils, as defined before, and other types of volatile extracts, obtained, for example, by washing the distillate water with a volatile solvent like dichloromethane. These other types of volatile extracts should be clearly defined and therefore not called “essential oils” as they do not strictly fall under this definition. Nevertheless, this review focuses on volatile compounds from lichens obtained by means of steam distillation, hydrodistillation, or by extraction using a Clevenger-type apparatus. The extract of interest can therefore be an essential oil, clearly distinguishable from the distillation water, or a fraction obtained by washing the distillation water with an organic solvent, as described by Sanad et al. [96], for example. The main benefit of applying this extraction procedure to lichens is that it selectively separates the volatile compounds with a molecular weight inferior to 300 Da and therefore enables the use of gas chromatography techniques for their analysis, without prior derivatization. This type of compound (volatile) seems to be relatively rare in lichens and their study is hindered by the fact that the majority of lichenic compounds are polar and present low volatility [65]. This review draws attention to the antimicrobial potential of essential oils and volatile compounds from lichens, a relatively untapped resource, in the already widely studied field of secondary metabolites and extracts from lichens with antimicrobial potential.

To the best of our knowledge, very few reports exist about essential oils and volatile compounds extracted from lichens. We have found, at the date of this review, only six articles on this subject [18,96,97,98,99,100]. Among these six articles, four investigated the antimicrobial potential of lichen volatile compounds [18,96,97,98], and provided chemical characterization by GC-MS, whereas the other two studies provided only the GC-MS chemical composition without investigating the antimicrobial potential [99,100]. We noticed that the article by Maqbul et al. [18] is unclear regarding the dose of essential oil or hydrolat used against the different pathogens. As mentioned previously, the essential oil and volatile compound extraction process using a Clevenger or hydrodistillation apparatus has an impact on the composition of the extract [94]. The heating process, associated with water or steam contact, is probably responsible for an array of chemical transformations which result in the unique composition of these extracts, and probably results in the presence of compounds not previously described in the organism of origin [94]. Moreover, the process of extraction also results in a narrower spectrum of compounds compared to classical extracts [94]. From a chemical point of view, the composition of these new types of extracts seems interesting.

The compilation of the exploitable data of GC-MS (Table 3) highlights the presence of 106 compounds in seven lichen species. The Kovats retention index (KI) ranges from 927 to 2900.

Lichens of the genus *Evernia* harvested from Posof, Ardahan, Turkey, demonstrated a quite similar composition [97], whereas the composition of two *Evernia prunastri* extracts from Turkey and Serbia were quite different. This is probably due to the difference in the protocols of extraction used. The Turkish *E. prunastri* extract is an essential oil, whereas the other is the acetone fraction of a methanolic extract [97,99]. The major compounds detected were γ-himachalene (37.51%) in *L. pygmae*, olivetol (33.5%) in *H. physodes*, atraric acid (30.3 and 30.1%) respectively in *P. sulcata* and *E. prunastri*, orcinol (25%) in *E. prunastri*, and α-tocopherol (24.7%) in *P. sulcata*.

In comparison with classical extracts, volatile compounds from lichens seem to be active against the tested microorganisms with low MICs (minimum inhibitory concentrations), generally less than 1 mg/mL (Table 4). They are more active against Gram-positive bacteria, as “classical” extracts are, and against fungi. Nevertheless, the scarcity of data on the subject makes it difficult to formulate assumptions and extract relevant conclusions.

We formulate in this review the hypothesis that hydrodistillation, steam distillation, and Clevenger-type distillation in water are interesting and novel ways of extracting compounds with a bioactive potential from lichens. The absence of previous in-depth work on the subject can be explained, in our opinion, by the relatively low yield of extraction (*E. prunastri* (0.32%) and *E. divaricata* (0.22%)) [97], in comparison with maceration or Soxhlet with organic solvents (around 2 to 10%). Another parameter to consider is the relatively low biomass available in the field, and the long regeneration time of these organisms [25,38]. Another limitation of this review is the potential for publication bias, as studies demonstrating potent antimicrobial activity from lichen volatiles are more likely to be published than those with null results. Furthermore, the scarcity of research in this specific niche may lead to an over-representation of certain prolific lichen genera, such as *Evernia* or *Cladonia*.

Despite these limitations, a more thorough investigation should be pursued in the field to unveil potential new molecules with high added value, such as antihuman pathogens and antiproliferative activities. This research should focus on the screening, identification, and fractionation of lichen volatile compounds. After these steps are completed, we suggest the development of hemi-synthesis of these potentially interesting molecules using organic molecules from organisms without these growth and regeneration limitations, or by integrating lichen genes in fast-growing organisms such as yeast [74]. This is particularly relevant because in vitro lichen resynthesis is extremely difficult and is not yet sufficiently efficient to yield enough biomass production in laboratory conditions [101,102].

## 5. Antimicrobial Potential of Lichen Extracts

Lichens are a unique reservoir of low-molecular-weight compounds with promising biological activities [86,103]. Their extracts display a wide range of activities, ranging from antimicrobial [7,86] to antifungal [104], antioxidant [103], and anticancer [7]. The first investigation focusing on the antimicrobial potential of lichen extracts dates to the 1940s and was probably motivated by the isolation of penicillin from a fungus [105]. Since then, a variety of extracts have been characterized by analytical methods and compounds isolated from numerous lichen species and investigated for their bioactivities [103]. The antimicrobial potential of lichen extracts seems overall to be promising against Gram-positive bacteria and yeasts of the genus *Candida*, while the effect of the extracts against Gram-negative bacteria is more anecdotal [8].

### 5.1. Evernia prunastri (L.) Ach. Extracts

*Evernia prunastri* (L.) Ach. is a lichen of the Parmeliaceae family characterized by a fruticose thallus, generally pendant and ramified. The upper surface containing the photobiont is generally yellowish-green to grayish-green and the underside whitish. The cortex generally contains usnic acid, atranorin, and chloroatranorin, and the medulla evernic acid. *Evernia prunastri* usually grows on neutral to acidic bark (stems, branches and twigs), especially of oaks and other broadleaf trees or shrubs (only occasionally on conifers), usually at lower elevations (but up to 1675 m) in areas with high humidity but mainly in sunny, often windswept zones. It has a very wide distribution; it is incompletely circumpolar. It can be found in western North America, Europe, northern Africa and Japan [106]. The extracts of this lichen are widely used in perfumery under the denomination of “Oakmoss absolute” and are one of the most sought-after lichen products [59]. Due to its relative abundance and ease of identification, it is one of the main studied lichens. The most notable biological properties of this lichen are antioxidant, antibacterial [14], antifungal [107], cytotoxic [108], anticancer [109], and anti-genotoxic [110]. Regarding the antimicrobial potential of extracts of this lichen (Table 5), the dichloromethane extract demonstrated the lowest MIC in *Staphylococcus aureus* (MIC = 4 µg/mL) [14].

The acetone extract is the most used, followed by the methanol extract. Other organic solvents are used, but more marginally, as is the case with acetonitrile, dichloromethane, hexane, and ethanol. Extracts of *E. prunastri* appear to be most potent against Gram-positive bacteria and *Candida* yeasts. The lowest potential is observed against Gram-negative bacteria.

### 5.2. Evernia divaricata (L.) Extracts

*Evernia divaricata* (L.) is a lichen of the Parmeliaceae family. It has a long fruticulose and pendant thallus. It is richly but irregularly branched. The color ranges from greenish gray to grayish yellowish green or pale yellowish green when fresh, often with irregularly elongated and branched maculae of a paler, more yellowish color than the rest, and the finer branches becoming gray-brown, darkest at the extreme tips. The cortex contains usnic acid, and the medulla divaricatic acid. *Evernia divaricata* grows on stems, branches, or twigs, mostly of conifers (especially spruce), in humid locations in montane to subalpine forests. It can be found in Europe, the Near East, China, and western North America [106]. Extracts of this lichen have shown antimicrobial [120], antioxidant [112], antiproliferative and apoptotic activities [121]. The most potent antibacterial activity of this species (Table 6) was demonstrated by the methanolic extract, with an MIC of 15.62 µg/mL, against *Klebsiella pneumonia*, *Proteus vulgaris*, *Pseudomonas aeruginosa*, and *Staphylococcus aureus* [112].

### 5.3. Cladonia Rangiformis Hoffm. Extracts

*Cladonia rangiformis* Hoffm. is a lichen of the Cladoniaceae family. It has a ramified fruticose thallus, with a round cross-section, and a squamulate shape. The surface appears spotted due to the uneven distribution of algal cells. The thallus contains atranorin, fumarprotocetraric acid, and rangiformic acid [124]. *Cladonia rangiformis* Hoffm. extracts have shown antimicrobial [125,126], antioxidant [127,128], anti-genotoxic [129], antimutagenic [130,131], anti-inflammatory [132], antiproliferative and apoptotic potential [133]. The most potent antibacterial extract (Table 7) was the chloroform extract, with an MIC value of 6 μg/mL against *Escherichia coli* and *Pseudomonas aeruginosa* [125], whereas the most potent antifungal was the cyclohexane extract with an MIC of 8 μg/mL against *Candida glabrata* [126].

### 5.4. Cladonia furcata (Huds.) Schrad Extracts

*Cladonia furcata* (Huds.) is a lichen of the Cladoniaceae family. The primary thallus is squamulose, usually disappearing. The squamules are up to 4 mm long and 3 mm wide, irregularly lobate to crenate–lobate. The podetia are 15 to 80 mm tall, 0.5–5 mm wide, pale, or bluish gray to dark brown, subulate, without cups or, occasionally, forming cup-like axils, dichotomously branched. The thallus contains fumarprotocetraric acid. It grows on soil or among mosses, rarely on rotting wood. It has a temperate distribution. It is present on all continents except Antarctica [106]. Extracts of *Cladonia furcata* have demonstrated antimicrobial [134,135,136], antioxidant [137,138], cytotoxic [139], anticancer [140,141], antiproliferative [115], and anti-phytopathogenic activities [142]. The acetone extract is the most investigated (Table 8), followed by the methanol extract and lastly the water extract.

The most potent extract is the acetone extract with an MIC of 0.39 mg/mL against *Bacillus subtilis* [134]. Atranorin is the most potent of the compounds isolated from this lichen species, with an MIC of 0.015 mg/mL against *Bacillus mycoides* [138].

### 5.5. Parmotrema perlatum (Huds) M. Choisy Extracts

*Parmotrema perlatum* (Hudson) M. Choisy is a lichen of the Parmeliaceae family. The thallus is foliose, adnate with a diameter ranging from 3 to 20 cm, and is lobate. The lobes are sub-irregular, elongated, often imbricate, and plane; they are separate and measure 4 to 15 mm in width. The upper surface is gray and smooth, sometimes with some blackened areas. The medulla is white, with a continuous algal layer. The lower surface is black with a peripheral brown naked zone. The upper cortex contains atranorin and chloroatranorin, while the medulla contains stictic acid (major), constictic acid (minor), and norstictic, menegazziaic and cryptostictic acids (traces). *Parmotrema perlatum* usually grows on hardwood trees in open habitats, occasionally on rocks. It is a widespread lichen found in temperate region of the Northern and Southern Hemispheres [106]. Its extracts have been investigated for biological activities such as antimicrobial [145,146,147], antioxidant [147], spasmolytic, bronchodilatory and vasodilatory [148]. The most frequently investigated solvents for this lichen species are hexane, followed by methanol, chloroform, ethanol, and water (Table 9).

A study by Devi et al. [149] has highlighted the potential antibacterial activity of *Parmotrema perlatum* n-hexane and methanolic extracts against some drug-resistant bacteria (Carbapenem-resistant *Pseudomonas aeruginosa* and *Klebsiella pneumoniae*, Cephalosporin-resistant *Clostridium difficile*, and Macrolide-resistant *Streptococcus pyogenes*), and against *Mycobacterium tuberculosis*. The composition of the extract has been assessed and is different between methanol and hexane. Moreover, the methanolic extract seems to be the most potent against all the tested bacteria, with an MIC of 185.10, 177.84, 186.28 and 202.60 μg/mL against *P. aeruginosa*, *K. pneumoniae*, *C. difficile*, and *S. pyogenes*, respectively. Regarding *Mycobacterium tuberculosis*, the methanolic extract demonstrated a 90% growth inhibition of the strain h37 Rv at 80 μg/mL. The major compounds of the methanolic extract include Stigmastan 3,5-diene, Gamolenic acid, Longidione, Platambin, and 5,7-dihydroxy 4-methyl coumarin.

## 6. Discussion and Perspectives

### 6.1. Mechanisms of Action of Lichen Volatiles Against Resistant Pathogens

The findings of this systematic review suggest that the volatile fraction of lichens could represent a chemically distinct antimicrobial resource, though it has often been overshadowed by “classical” secondary metabolites such as usnic acid and atranorin (detailed in Section 5). While traditional solvent extracts (acetone/methanol) frequently rely on larger, relatively immobile molecules, the essential oils and VOCs identified in this study, rich in low-molecular-weight phenolics and sesquiterpenes, appear to offer certain pharmacological advantages. Their characteristic volatility and lipophilicity may indicate a potential that likely complements, rather than merely replicates, the activity of traditional extracts. This represents a direction of research that remains relatively untapped, potentially due to the practical challenges associated with biomass constraints. While the primary studies included in this review focused on standard ATCC strains, the chemical profiles identified, rich in atraric acid, orcinol, and sesquiterpenes, point toward specific mechanisms that are highly relevant to overcoming multidrug resistance. Unlike traditional antibiotics that often target a single protein, lichen volatiles appear to exert multi-target effects [150]:

- Membrane Permeabilization: The high concentration of phenolic volatiles, such as atraric acid and methyl haematommate, suggests a mechanism involving the disruption of the bacterial lipid bilayer. Phenolic compounds are known to integrate into the cell membrane, altering its fluidity and increasing permeability, which leads to the leakage of essential intracellular components [151]. This “leakage” effect is particularly effective against Gram-positive bacteria and can serve as a synergistic tool to help conventional antibiotics bypass the physical barriers of resistant strains [152].
- Efflux Pump Interference: Several sesquiterpenes identified in species like *Lichina pygmaea* (e.g., γ-himachalene) belong to chemical classes known to act as efflux pump inhibitors. By blocking the proteins that bacteria use to “spit out” drug molecules, these volatiles could potentially restore the efficacy of currently obsolete antibiotics [153].
- Biofilm Disruption: Volatile organic compounds (VOCs) are known to interfere with Quorum Sensing (QS), the chemical signaling bacteria use to coordinate the formation of protective biofilms [154]. Since biofilms represent a primary defense mechanism for MDR pathogens, disrupting these structures is a critical strategy in treating chronic and persistent infections.

### 6.2. Sustainability and the “Biomass Bottleneck”

As noted in our findings, the extraction yields for lichen essential oils are significantly lower (generally <1% *w*/*w*) than those of traditional organic solvent extracts, which can reach 10% *w*/*w* or more. This disparity creates a profound sustainability challenge. Lichens are notorious for their slow growth rates, often measured in millimeters per year, and their complex symbiotic nature makes large-scale cultivation in bioreactors extremely difficult [102]. Consequently, harvesting massive quantities of wild lichen biomass to extract trace amounts of volatiles is ecologically unviable and poses a threat to biodiversity. Therefore, lichen antimicrobial potential should not be viewed as a resource to be exploited through direct bulk extraction, but rather as a chemical blueprint for the development of synthetic or semi-synthetic analogs.

### 6.3. Future Directions: From Thallus to Lab

The transition of lichen volatiles into the therapeutic arsenal requires moving away from wild harvesting toward laboratory-based production. Two primary strategies emerge as the most viable:

- Heterologous Expression: Recent advances in lichen genomics have identified specific polyketide synthase (PKS) gene clusters responsible for the biosynthesis of phenolic volatiles like atraric acid and orsellinic acid derivatives [66]. By integrating these genes into fast-growing, easily cultivable heterologous hosts such as *Saccharomyces cerevisiae* or *Escherichia coli*, researchers can produce specific lichen metabolites at scale without the need for thallus biomass [74].
- Hemi-synthesis and Biomimetic Synthesis: Many lichen volatiles possess a relatively simple phenolic core. Future pharmaceutical development could utilize more abundant, plant-derived phenolics (such as resorcinol or orcinol derivatives) as scaffolds. These can be chemically modified through esterification or halogenation to “mimic” the unique structures found in lichens, such as chloroatranol or methyl haematommate, creating a sustainable supply of lead compounds for drug testing [60].

## 7. Conclusions

This systematic review identifies lichen-derived volatile compounds and essential oils as a promising, yet critically under-researched, antimicrobial resource. Based on our qualitative synthesis, the certainty of evidence is considered moderate for Gram-positive pathogens, where consistent growth inhibition was observed across independent reports. However, the certainty remains low for Gram-negative bacteria and clinical multidrug-resistant (MDR) isolates, primarily due to the limited number of studies (*n* = 6), high chemical variability, and a current reliance on standard laboratory strains.

To transition these “hidden” metabolites into the therapeutic pipeline, a paradigm shift is required. Future research must move beyond the preliminary screening of wild-harvested thalli toward the evaluation of pure lead compounds against resistant clinical phenotypes. Furthermore, while this review focused on antimicrobial potential, the total lack of data regarding the cytotoxic, antidiabetic, or neuroprotective effects of these volatile fractions represents a significant missed opportunity for drug discovery.

Ultimately, the sustainability of this field depends on decoupling research from biomass consumption. By adopting the laboratory-based production strategies outlined in this review, namely biosynthetic engineering and biomimetic synthesis, researchers can explore the full pharmaceutical potential of lichen volatiles without compromising the biodiversity of these slow-growing organisms.

## Figures and Tables

**Table 1:** General classification of the different phenotypes of lichen-forming fungi. Adapted from Honegger [38].

Thallus Morphology		Description		Examples
Gelatinous lichens		Lack of aerial hyphae systems with hydrophobic cell wall surfaces. The gelatinous extracellular sheaths swell in the presence of water and shrink in dry conditions.		Generally cyanolichens: Collemataceae, Peltigerales, *Lichina* spp.
				Sometimes chlorolichens: *Epigloea*, *Thrombium*
Microfilamentous lichens		Derived morphology from overgrown, ensheeted filamentous photobiont similar to a filamentous alga.		Cyanolichens: *Pyrenothrix nigra*, *Ephebe lanata* (L.) Vain.
				Chlorolichens: *Cystocoleus ebeneus* (Dillwyn) Thwaites, *Racodium rupestre* Pers., *Coenogonium* spp., *Psoroglaena* spp.
Microglobose lichens		Small globules with peripheral cortex containing photobiont cells.		Chlorolichens: *Micarea* spp., *Vezdaea* spp.
Leprose lichens		Powdery crusts over natural and anthropogenic surfaces, built up by loosely interwoven hyphae with very hydrophobic wall surfaces overgrowing groups of photobiont cells. Sterile; dispersion by fragmentation.		*Lepraria*, *Leproloma*, *Leprocaulon* spp., *Chrysothrix* spp.
Crustose lichens		The most common and widespread symbiotic phenotypes among all lichenized fungi groups. Crustose thalli are homoeomerous, neither internally stratified nor differentiating a peripheral cortex. Sometimes Endolithic or Endophloeodal; generally, they form either tiny areoles or thick crustose thalli without internal stratification.		50% of lichen-forming ascomycetes. *Verrucaria* spp., *Bagliettoa* spp., *Patella* spp.
Placodioid lichens		Presence of a cortical layer and an internal stratification; adhere to the substratum with their entire lower surface and are areolate at their periphery.		*Ophioparma ventosa* (L.) Norman (Ov), *Xanthoria elegans* (Link.) Th. Fr.
Squamulose lichens		Presence of a cortical layer and an internal stratification; small, dorsiventrally organized scales with an upper cortex and an algal layer, but usually without lower cortex.		*Endocarpon pusillum* Hedw.
Macrolichens	Foliose lichens	Dorsiventrally organized, all with internal stratification	Foliose structure (leaf-like), or umbilicate (leaf-like with central holdfast).	*Peltigera malacea*, *Peltigera britannica*, *Xanthoria* *parietina* (L.) Th. Fr., *Parmelia sulcata* Taylor, *Lasallia pustulata* (L.) Mérat
	Fruticose lichens		Band-shaped thalli and fruticose (shrubby), either erect or pendulous morphotypes. May form large amounts of biomass on restricted grounds.	*Bryoria*, *Usnea and Ramalina* spp. among Lecanorales, *Teloschistes* spp. among Teloschistales. *Cladonia rangiferina* (L.) F.H. Wigg.

**Table 2:** Biosynthetic pathways and biological properties of some lichenic acids.

Compound	Biosynthetic Pathway	Biological Activities	Toxicity	Allergenic Potential
Atranorin	Polyketide synthase gene cluster identified in *Stereocolon alpinum.* Three enzymes intervene: a polyketide synthase, a cytochrome P450 monooxygenase and a O-methyl-transferase [66,67]. Gene cluster has already been heterologously expressed in the phytopathogenic fungi *Ascochyta rabiei* [67].	Analgesic, anti-inflammatory, antiulcer, antidiabetic, antioxidant, cytotoxic, antimicrobial, antifungal, antiviral, antiparasitic, larvicidal, and potential neuroprotective activities [8].	Acute and sub-chronic toxicity assessment of this compound in rodents: 5 g/kg single dose of bodyweight for 14 days and 50 mg/kg of bodyweight per day for 30 days, respectively. No major adverse effects at the doses tested [68].	Potential allergen, allergenic properties potentially amplified by sunlight [68,69]
Usnic acid	Polyketide synthase gene cluster identified in *Cladonia uncialis* [70]. At least two enzymes are necessary for its biosynthesis, a polyketide synthase and a cytochrome P450 [66].	Anti-inflammatory, antibacterial, antiviral, antitumor, antioxidant, photoprotection and wound-healing properties [71].	In vitro and in vivo toxicity studies revealed its hepatotoxicity, through mechanisms involving uncoupling of oxidative phosphorylation and production of free radicals [72]. This molecule seems not to be genotoxic [72].	Potential skin sensitizer and allergen [69]
Gyrophoric acid	Exact biosynthetic pathway yet to be determined, but it is highly likely that it involves the genes that are contained on the PKS16 gene cluster [66,73].	Antimicrobial, antioxidant, cytotoxic, photoprotective, larvicidal and antihypertensive agent [8].	- ^1^	-
Lecanoric acid	Biosynthetic pathway involves a non-reducing polyketide synthase gene cluster identified as PFUR17_02294 and heterologously expressed in *Saccharmyces cervisae* [74].	Antimicrobial, anthelmintic, antioxidant and anticancer properties [8,75,76,77].	-	-
Oliveteric acid	Polyketide synthase involved in its biosynthesis [66,78].	Antioxidant and cytotoxic activities reported [79,80,81].	-	-
Physodic acid	Polyketide synthase is involved in the biosynthesis, additionally requiring the cytochrome P450 [66,78].	Antioxidant and cytotoxic activities reported [79,80,81].	-	-

^1^ No data found.

**Table 3:** Chemical composition of the essential oils and volatile compounds from various lichens, analyzed by gas chromatography–mass spectrometry (GC-MS) (t = trace, KI = Kovats retention index, %Area = relative abundance in % of total volatile compounds). Adapted from [86,87,88,89].

Compounds	KI	% Area							
		*E. divaricata*	*E. prunastri*	*E. prunastri*	*P. sulcata*	*H. physodes*	*C. rangiformis*	*C. furcata*	*L. pygmaea*
2,3-dibutyloxirane	-	-	-	-	-	-	-	-	1.14
Tricyclene	927	2.2	0.5	-	-	-	-	-	-
α-Pinene	939	7.2	6.6	-	-	-	0.9	0.2	-
Camphene	954	3.1	3.0	-	-	-	-	-	-
β-Pinene	979	8.0	6.3	-	-	-	-	-	-
1-Octen-3-ol	979	-	-	-	-	-	15.7	15.7	-
3-Octanone	984	-	-	-	-	-	21.7	18.6	-
3-Octanol	991	-	-	-	-	-	11.7	-	-
2-Pentyl furan	993	-	1.7	-	-	-	-	-	-
α-Phellandrene	1003	4.1	3.3	-	-	-	-	-	-
α-Campholenal	1126	1.8	-	-	-	-	-	-	-
Limonene	1029	6.3	1.6	-	-	-	5.4	-	-
Benzene acetaldehyde	1042	-	-	-	-	-	2.0	0.4	-
γ-Terpinene	1060	1.9	0.5	-	-	-	-	-	-
Terpinolene	1089	3.1	-	-	-	-	-	-	-
p-Cymene	1091	1.8	1.5	-	-	-	-	-	-
Nonanal	1101	-	-	-	-	-	1.9	-	-
trans-Pinocarveol	1139	2.0	2.7	-	-	-	-	-	-
1-Nonanol	1169	-	-	-	-	-	2.0	-	-
Decanal	1202	-	-	-	-	-	1.9	-	-
trans-Carveol	1217	1.8	-	-	-	-	-	-	-
Carvone	1243	2.2	-	-	-	-	-	-	-
1-decanol	-	-	-	-	-	-	-	-	0.76
(*Z*)-2-decenal	-	-	-	-	-	-	-	-	3.13
(*E*)-2-decenal	-	-	-	-	-	-	-	-	0.3
α-Terpinen-7-al	1285	2.9	2.6	-	-	-	-	-	-
Bornyl acetate	1289	2.5	1.7	-	-	-	-	-	-
2-Undecanone	1294	1.7	-	-	-	-	-	-	-
(*E*,*E*)-2,4-decadienal	-	-	-	-	-	-	-	-	4.1
(*2E*,*4E*)-2,4-decadienal	-	0.6	0.3	-	-	-	-	-	8.59
(*E*)-2-pentenol	-	-	-	-	-	-	-	-	0.35
Orcinol monomethyl Ether	-	-	-	5.7	-	t	-	-	-
Limonene oxide	-	-	-	-	-	-	-	-	0.43
2-undecenal	-	-	-	-	-	-	-	-	2.71
Orcinol	-	-	-	25.0	t	0.6	-	-	-
α-Copaene	1377	2.5	1.0	-	-	-	-	-	-
(*E*)-4,5-epoxy-2-decenal	-	-	-	-	-	-	-	-	2.3
Tetradecane	1400	-	-	-	-	-	0.5	-	-
Longifolene	1408	-	-	-	-	-	0.5	-	-
(Z)-Caryophyllene	1409	0.6	-	-	-	-	-	-	-
(E)-Caryophyllene	1419	2.8	-	-	-	-	0.5	-	-
α-Humulene	1455	1.4	1.2	-	-	-	-	-	-
*α*-longipinene	-	-	-	-	-	-	-	-	0.31
*ς*-muurolene	-	-	-	-	-	-	-	-	0.31
*E-β*-farnesene	-	-	-	-	-	-	-	-	1.45
1-methyl-4-(6-methylheptan-2-yl)benzene	-	-	-	-	-	-	-	-	0.93
4,5-di-epi-aristolochene	-	-	-	-	-	-	-	-	0.36
1-Pentadecene	1493	-	-	-	-	-	1.1	-	-
Chloroatranol	-	-	-	-	-	0.5	-	-	-
Pentadecane	1500	-	-	-	-	-	-	0.3	-
α-Muurolene	1500	1.4	1.8	-	-	-	-	-	-
(*R*)-cuparene	-	-	-	-	-	-	-	-	0.61
δ-Amorphene	1512	0.8	-	-	-	-	-	-	-
Selina-3,7(11)-diene	1547	-	-	-	-	-	1.0	-	-
Atranol	-	-	-	2.1	5.2	5.1	-	-	-
Sparassol	-	-	-	1.6	-	-	-	-	-
Caryophyllene oxide	1583	-	-	-	-	-	2.4	-	-
Hexadecane	1600	-	-	-	-	-	-	1.0	-
Tetradecanal	1613	-	-	-	-	-	1.0	-	-
Methyl orsellinate	-	-	-	10.2	-	-	-	-	-
*α*-himachalene	-	-	-	-	-	-	-	-	7.62
(E)-Citronellyl tiglate	1668	2.8	7.8	-	-	-	1.7	10.7	-
Methyl haematommate	-	-	-	3.4	1.9	1.5	-	-	-
8-Heptadecene	1670	-	-	-	-	-	1.7	18.3	-
1-Heptadecene	1693	-	-	-	-	-	2.7	-	-
Acorenone B	1698	-	-	-	-	-	1.2	-	-
Heptadecane	1700	2.9	1.2	-	-	-	-	-	-
Methyl β-orcinolcarboxylate (atraric acid)	-	-	11.5	30.1	30.3	17.2	3.8	2.5	-
*γ*-himachalene	-	-	-	-	-	-	-	-	37.51
*β*-himachalene	-	-	-	-	-	-	-	-	11.71
1,3,5-himachalatriene	-	-	-	-	-	-	-	-	3.25
Naphthalene	-	-	-	-	-	-	-	-	1.77
Orsellinic acid	-	-	-	1.1	-	-	-	-	-
Olivetol	-	-	-	-	1.6	33.5	-	-	-
Guaiazulene	-	-	-	-	-	-	-	-	1.26
1-Octadecene	1790	-	-	-	-	-	1.0	-	-
Diisobutyl phtalate	1869	-	6.5	-	-	-	-	-	-
Caryophyllene oxide	1583	-	2.6	-	-	-	-	-	0.35
Nonadecane	1900	1.5	-	-	-	-	-	-	-
Cyclohexadecanolide	1935	-	-	-	-	-	0.8	-	-
Palmitic acid	-	-	-	t	2.1	0.9	-	-	-
Eicosane	2000	-	0.7	-	-	-	-	-	-
Epi-13-manoyl oxide	2017	-	2.4	-	-	-	-	-	-
Abietatriene	2057	0.9	1.3	-	-	-	-	-	-
Heneicosane	2100	1.5	1.8	-	-	-	-	-	-
Linoleic acid	-	-	-	0.9	2.1	0.6	-	-	-
α-linolenic acid	-	-	-	-	3.3	-	-	-	-
Oleic acid	-	-	-	t	3.5	3.2	-	-	-
Stearic acid	-	-	-	t	0.7	0.9	-	-	-
1-Docosene	2190	1.3	3.4	-	-	-	-	-	-
Docosane	2200	1.3	-	-	-	-	-	6.4	-
Cadalene	-	-	-	-	-	-	-	-	1.72
Olivetonide	-	-	-	-	-	15.7	-	-	-
1-Tricosene	2296	2.5	10.1	-	-	-	-	-	-
Tricosane	2300	-	4.3	-	-	-	-	14.6	-
Olivetonic acid	-	-	-	-	-	7.7	-	-	-
Tetracosane	2400	1.6	-	-	-	-	5.1	-	-
Pentacosane	2500	2.1	0.5	-	-	-	1.0	17.0	-
Usnic acid	-	-	-	11.4	-	-	-	-	-
Nonacosane	2900	-	-	-	2.4	-	-	-	-
n-hexadecanoic acid	-	-	-	-	-	-	-	-	0.57
α-tocopherol	-	-	-	-	24.7	0.6	-	-	-
Lichesterol	-	-	-	t	1.8	1.3	-	-	-
Ergosterol	-	-	-	t	4.5	2.1	-	-	-
β-sitosterol	-	-	-	-	10.6	-	-	-	-
Total (% area)		81.1	90.4	91.5	91.4	91.4	89.2	91.6	93.54
Type of extract		Essential oils			Volatile compounds (acetone fraction of a methanol extract)	Essential oils			Volatile compounds (dichloromethane fraction of hydrolat)
Reference			[97]		[99]		[98]		[96]

**Table 4:** Antimicrobial potential of essential oils and volatile compounds from various lichen species.

Species	Harvest Location	Type of Compound	Target Microorganism	MIC Values/ZI Diameters	Composition	References
*Evernia prunastri* (L.) Ach.	Posof, Ardahan-Turkey	Essential oil	*Candida albicans*	15.6 µg/mL	Monoterpenes hydrocarbons (23.3%) and oxygenated monoterpenes (13.0%). The major compounds are: β-pinene (6.3%), α-pinene (6.6%), limonene (1.6%), α-phellandrene (3.3%), camphene (3.0%) and ρ-cymene (1.5%).	[97]
*Evernia divaricata* (L.) Ach.		Essential oil	*Escherichia coli* *Yersinia pseudotuberculosis* *Staphylococcus aureus* *Entercoccus faecalis* *Bacillus cereus*	471.9 µg/mL 943.7 µg/mL 235.9 µg/mL 235.9 µg/mL 943.7 µg/mL	Monoterpenes hydrocarbons (37.7%) and oxygenated monoterpenes (13.0%). The major compounds are: β-pinene (8.0%), α-pinene (7.2%), limonene (6.3%), α-phellandrene (4.4%), camphene (3.1%) and ρ-cymene (1.8%).	
*Parmotrema perlatum*	Not specified	Essential oil	*Streptococcus* sp. *Staphylococcus* sp. *Escherichia* sp. *Pseudomonas* sp.	Expressed in dL/mL	Alkaloids (0.6%), carbohydrates (1.9%), phytosterols (1%), fixed oils and fats (5.2%), phenolic compounds (0.2%), tannins (1.3%), proteins (0.3%), amino acids (38.1%), gums and mucilage (0.1%), saponin (0.1%), carvacrol (2.3%), caryophyllene (3.1%).	[18]
*Lichina pygmaea* (Lightf.) C. Agardh	Rocky shores of El Jadida, Atlantic coast of Morocco.	Volatile compounds	*Staphylococcus aureus* *Pseudomonas aeruginosa* *Escherichia coli* *Candida albicans*	13.5 mg/mL 13.5 mg/mL 1.69 mg/mL 13.5 mg/mL	Twenty-five volatile organic constituents which represent 93.54% of total volatile compounds. Sesquiterpenes was the major class of compounds. The major compounds are γ-himachalene (37.51%), β-himachalene (11.71%), (2E,4E)-2,4-decadienal (8.59%) and α-himachalene (7.62%).	[96]
*Cladonia rangiformis* Hoffm.	Posof, Ardahan, Turkey	Essential oil	*Entercoccus faecalis* *Candida albicans*	306.2 µg/mL 306.2 µg/mL	Twenty-five components, which constituted 89.2% of the oil, were identified. The classes of compounds were monoterpene hydrocarbons (6.3%), sesquiterpene hydrocarbons (2.0%), oxygenated sesquiterpenes (3.6%), terpene-related compounds (1.7%), hydrocarbons (13.1%), alcohols (29.4%), aldehydes (6.8%), esters (7.9%), and ketones (21.7%). The main compounds were 3-octanone (21.7%), 1-octen-3-ol (15.7%), 3-octanol (11.7%), limonene (5.4%) and tetracosane (5.1%)	[98]
*Cladonia furcata* (Huds.) Schrad.	Posof, Ardahan, Turkey	Essential oil	*Candida albicans*	784.4 µg/mL	Twelve components, which constituted 91.6% of the oil, were identified. The classes of compounds were monoterpene hydrocarbons (0.2%), terpene-related compounds (10.7%), hydrocarbons (57.6%), alcohols (1.6%), aldehydes (0.4%), esters (5.9%), and ketones (18.6%). The main compounds were 3-Octanone (18.6%), 8-heptadecene (18.3%), pentacosane (17.0%), tricosane (14.6%), and (E)-citronellyl tiglate (10.7%).	

**Table 5:** Antimicrobial activity of extracts of the lichen *Evernia prunastri* (L.) Ach. from different localities.

Type of Extract	Harvest Location	Target Microorganism	MIC Values/ZI Diameters	Extract Composition	References
Methanol macerate	Khenifra, Morocco	*Staphylococcus aureus* ^1^	from 0.07 to 0.15 mg/mL	Methyl lecanorate, evernic acid, usnic acid, atranorin and chloroatranorin (evernic acid was the most abundant compound).	[111]
Acetone macerate	Khenifra, Morocco	*Bacillus subtilis* *Listeria innocua* *Staphylococcus aureus* *Escherichia coli* *Pseudomonas aeruginosa* *Proteus mirabilis*	0.078 mg/mL 0.625 mg/mL 0.078 mg/mL >25 mg/mL >25 mg/mL >25 mg/mL	Evernic acid, usnic acid, atranorin, and chloratranorin were identified, with evernic acid being the most abundant.	[108]
Methanolic extract	Artvin Province, Turkey	*Clavibacter michiganense* *Escherichia coli* *Pseudomonas syringae*. pv. *tomato* *Streptococcus pyogenes* *Xanthomonas campestris* *Aspergillus niger* *Penicillum* spp. *Sclorotinia sclerotiorum*	15.62 µg/mL 15.62 µg/mL 31.25 µg/mL 15.62 µg/mL 31.25 µg/mL 62.50 µg/mL 62.50 µg/mL 31.25 µg/mL	N/A ^2^	[112]
Ethanolic extract	Bursa, Uludag, Turkey	*Aspergillus niger* *Penicillium expansum* *Botrytis cinerea* *Fusarium culmorum* *Fusarium solani* *Macrophomina phaseolina* *Rhizoctonia solani*	Other tests conducted ^3^	N/A	[107]
Acetone macerate	Cejkov, Slovakia	*Staphylococcus aureus* *Escherichia coli*	RIZD ^4^ (S.a) = 62.86% RIZD (E.c) = N/A Reference = gentamicin sulfate (50 µg/mL)	Salazinic acid, lecanoric acid, tetrahydroxy–tricosanoic acid, evernic acid, physodic acid, usnic acid, atranorin, chloratranorin and dihydrovinapraesoredisoic acid.	[113]
Acetone extract	Kopaonik, Serbia	*Bacillus mycoides* *Bacillus subtilis* *Escherichia coli* *Klebsiella pneumoniae* *Staphylococcus aureus* *Aspergillus flavus* *Aspergillus fumigatus* *Candida albicans* *Penicillium purpurescens* *Penicillium verrucosum*	6.25 mg/mL 6.25 mg/mL 25 mg/mL 6.25 mg/mL 12.5 mg/mL 12.5 mg/mL 12.5 mg/mL 6.25 mg/mL 25 mg/mL 12.5 mg/mL	Evernic acid, atranorin, chloroatranorin, physodic acid and usnic acid.	[109]
Acetone extract	Countryside around Limoges, France	*Candida albicans*	>100 µg/mL	Usnic and evernic acid.	[114]
Methanol extract	Bojanine Vode, Southeast of Serbia	*Sarcina lutea* *Enterococcus faecalis* *Enterococcus faecalis* *Bacillus subtilis* *Bacillus cereus* *Staphylococcus aureus* *Escherichia coli* *Pseudomonas aeruginosa* *Proteus mirabilis* *Salmonella enterica* *Salmonella typhymirium* *Candida albicans* *Rhodotorula* sp. *Saccharomyces boulardii* *Penicillium italicum* *Penicillium chrysogenum* *Penicillium digitatum* *Botrytis cinerea* *Trichothecium roseum* *Aspergillus niger* *Aspergillus niger* *Aspergillus restrictus* *Aspergillus fumigatus* *Aspergillus flavus*	7.81 × 10^−2^ mg/mL 10 mg/mL 3.13 × 10^−1^ mg/mL 3.91 × 10^−2^ mg/mL 7.81 × 10^−2^ mg/mL 1.56 × 10^−1^ mg/mL 10 mg/mL 2.5 mg/mL 5 mg/mL 10 mg/mL 10 mg/mL 2.5 mg/mL 1.25 mg/mL 5 mg/mL 1.25 mg/mL 1.25 mg/mL 1.25 mg/mL 1.25 mg/mL 1.25 mg/mL 1.56 × 10^−1^ mg/mL 1.25 mg/mL 1.25 mg/mL 1.25 mg/mL 1.25 mg/mL	N/A	[115]
Methanol extract	Tavush Province, Armenia	*Bacillus subtilis* *Staphylococcus aureus* *Pseudomonas aeruginosa* *Salmonella typhymurium*	3.75 mg/mL 3.75 mg/mL >7.5 mg/mL >7.5 mg/mL	N/A	[116]
Hexane extract	Mari El Republic, Russian Federation	*Staphylococcus aureus* *Pseudomonas aeruginosa* *Escherichia coli* *Candida albicans*	21 µg/mL 150 µg/mL >500 µg/mL 150 µg/mL	N/A	[14]
Dichloromethane extract		*Staphylococcus aureus* *Pseudomonas aeruginosa* *Escherichia coli* *Candida albicans*	4 µg/mL 167 µg/mL 500 µg/mL 150 µg/mL		
Acetonitrile 60% extract		*Staphylococcus aureus* *Pseudomonas aeruginosa* *Escherichia coli* *Candida albicans*	14 µg/mL 133 µg/mL 250 µg/mL 38 µg/mL		
Acetonitrile 60% extract—fraction n°5		*Staphylococcus aureus* *Pseudomonas aeruginosa* *Escherichia coli* *Candida albicans*	1.95 µg/mL 31.25 µg/mL 31.25 µg/mL 62.5 µg/mL	N/A	
Acetonitrile 60% extract—fraction n°6		*Staphylococcus aureus* *Pseudomonas aeruginosa* *Escherichia coli* *Candida albicans*	0.98 µg/mL 31.25 µg/mL 125 µg/mL 62.5 µg/mL	Evernic acid.	
Acetonitrile 60% extract—fraction n°7		*Staphylococcus aureus* *Pseudomonas aeruginosa* *Escherichia coli* *Candida albicans*	1.95 µg/mL >500 µg/mL >500 µg/mL 31.25 µg/mL	N/A	
Acetonitrile 60% extract—fraction n°8		*Staphylococcus aureus* *Pseudomonas aeruginosa* *Escherichia coli* *Candida albicans*	0.49 µg/mL >500 µg/mL >500 µg/mL 62.5 µg/mL		
Acetone extract	Dereli, Giresun, Turkey	*Staphylococcus calmii* *Bacillus pumilis* *Bacillus megaterium* *Acinetobacter baumannii* *Enterococcus faecium*	1000 µg/mL 500 µg/mL 500 µg/mL 500 µg/mL 62.5 µg/mL	N/A	[117]
Acetone extract	Bolu, Turkey	*Staphylococcus aureus* *Staphylococcus epidermidis* *Streptococcus pyogenes* *Proteus vulgaris*	16.0 ± 0.6 mm 23.5 ± 0.5 mm 25.0 ± 1.3 mm 10.0 ± 0.0 mm	Atranorin, evernic acid and usnic acid.	[118]
Methanol extract	Bolu, Turkey	*Staphylococcus aureus* *Staphylococcus epidermidis* *Streptococcus pyogenes* *Serratia marescens* *Proteus vulgaris*	16.3 ± 0.3 mm 24.5 ± 0.3 mm 22.75 ± 0.9 mm 7.5 ± 0.3 mm 11.5 ± 0.3 mm	Atranorin, evernic acid and usnic acid.	
Methanol extract	Bolu, Turkey	*Aeromonas hydrophila* *Streptococcus agalactiae* *Eterococcus faecalis* *Lactococcus garvieae*	21.3 ± 0.3 mm 16.0 ± 0.5 mm 12.0 ± 0.0 mm 17.0 ± 0.0 mm	N/A	[119]
Acetone extract	Bolu, Turkey	*Aeromonas hydrophila* *Streptococcus agalactiae* *Eterococcus faecalis* *Lactococcus garvieae*	24.5 ± 0.3 mm 16.0 ± 0.5 mm 15.0 ± 0.0 mm 16.0 ± 0.0 mm	N/A	

^1^ Eight strains of *S. aureus* investigated (ATCC25923 and 7 clinical isolates); ^2^ N/A: data not available; 3 test of inhibition of mycelia and spore growth; ^4^ %RIZD=[(IZD sample−IZD negative control)/IZD gentamicin] × 100, where RIZD is the relative inhibition zone diameter (%) and IZD is the inhibition zone diameter (mm). As a negative control, the inhibition zones of 5% DMSO equal to 0 were taken. The inhibition zone diameter (IZD) was obtained by measuring the diameter of the transparent zone.

**Table 6:** Antimicrobial activity of various extracts of the lichen *Evernia divaricata* (L.) Ach from different localities.

Type of Extract	Harvest Location	Target Microorganism	MIC Values/ZI Diameters	Extract Composition	References
Methanol extracts	Artvin Province, Turkey	*Acinetobacter baumanii* *Bacillus macerans* *Bacillus megaterium* *Bacillus subtilis* *Brucella abortus* *Clavibacter michiganense* *Enterobacter cloacae* *Enterococcus faecalis* *Escherichia coli* *Klebsiella pneumoniae* *Proteus vulgaris* *Pseudomonas aeruginosa* *Pseudomonas syringae*. pv. *tomato* *Salmonella enteritidis* *Staphylococcus aureus* *Streptococcus pyogenes* *Xanthomonas campestris* *Fusarium acuminatum* *Microsporum canis* *Rhizoctonia solani* *Sclorotinia sclerotiorum*	31.25 µg/mL 31.25 µg/mL 31.25 µg/mL 62.5 µg/mL 31.25 µg/mL 62.5 µg/mL 62.5 µg/mL 31.25 µg/mL 62.5 µg/mL 15.62 µg/mL 15.62 µg/mL 15.62 µg/mL 31.25 µg/mL 31.25 µg/mL 15.62 µg/mL 31.25 µg/mL 62.5 µg/mL 62.5 µg/mL 31.25 µg/mL 31.25 µg/mL 125 µg/mL	N/A ^1^	[112]
Acetone extract	Kastamonu Province, Turkey	*Bacillus toyonensis* *Bacillus mojavensis* *Bacillus amyloliquefaciens* *Bacillus subtilis* *Bacillus cereus* *Bacillus velezensis* *Bacillus licheniformis*	60 µg/mL 240 µg/mL 60 µg/mL 240 µg/mL ≥240 µg/mL ≥240 µg/mL ≥240 µg/mL	N/A	[122]
Acetone extract	Kastamonu Province, Turkey	*Enterococcus durans*	≥240 µg/mL	N/A	[123]
Methanol, aqueous and acetone extracts	Lake Golcuk, Bolu Province, Turkey	*Aeromonas hydrophila* *Aeromonas salmonicida* *Yersinia ruckeri* *Enterococcus faecalis* *Lactococcus garvieae* *Streptococcus agalactiae*	N/A N/A N/A N/A N/A N/A	N/A	[119]

^1^ N/A: data not available.

**Table 7:** Antimicrobial activity of extracts of the lichen *Cladonia rangiformis* Hoffm. from various localities.

Type of Extract	Harvest Location	Target Microorganism	MIC Values/ZI Diameters	Extract Composition	References
Methanol extract	Kandira District, Kocaeli Province, East Marmara Region, Turkey	*Escherichia coli* *Pseudomonas aeruginosa* *Enterococcus faecalis* *Staphylococcus aureus* *Candida albicans*	N/A N/A N/A N/A 161 μg/mL	N/A ^1^	[125]
Chloroform extract		*Escherichia coli* *Pseudomonas aeruginosa* *Enterococcus faecalis* *Staphylococcus aureus* *Candida albicans*	6 μg/mL 6 μg/mL N/A 8.4 μg/mL 9.6 μg/mL		
Cyclohexane extract	Cascade des vautours, massif Edough, Algeria	*Candida albicans* *Candida glabrata* *Aspergillus fumigatus* *Staphylococcus aureus* *Escherichia coli*	16 µg/mL 8 µg/mL >250 µg/mL 100 µg/mL >100 µg/mL	Isorangiformic acid, rangiformic acid, traces of usnic acid, atranorin, roccellic, jackinic, squamatic and evernic acids	[126]
Dichloromethane/methanol extract		*Candida albicans* *Candida glabrata* *Aspergillus fumigatus* *Staphylococcus aureus* *Escherichia coli*	>250 µg/mL >250 µg/mL >250 µg/mL 100 µg/mL >100 µg/mL		
Methanol/water extract		*Candida albicans* *Candida glabrata* *Aspergillus fumigatus* *Staphylococcus aureus* *Escherichia coli*	>250 µg/mL >250 µg/mL >250 µg/mL >100 µg/mL >100 µg/mL		

^1^ N/A: data not available.

**Table 8:** Antimicrobial activity of extracts of the lichen *Cladonia furcata* (Huds.) Schrad from various localities.

Type of Extract	Harvest Location	Target Microorganism	MIC Values/ZI Diameters	Extract Composition	Reference
Acetone extract	Kopaonik, Serbia	*Bacillus mycoides* *Bacillus subtilis* *Staphylococcus aureus* *Enterobacter cloaceae* *Escherichia coli* *Klebsiella pneumoniae* *Aspergillus flavus* *Aspergillus fumigatus* *Botrytis cinerea* *Candida albicans* *Fusarium oxysporum* *Mucor mucedo* *Paecilomyces variotii* *Penicillium purpurescens* *Penicillium verrucosum* *Trichoderma harsianum*	0.78 mg/mL 0.78 mg/mL 0.78 mg/mL 0.78 mg/mL 1.56 mg/mL 0.78 mg/mL 25 mg/mL 12.5 mg/mL 25 mg/mL 6.25 mg/mL 25 mg/mL 25 mg/mL 12.5 mg/mL 25 mg/mL 25 mg/mL 25 mg/mL	N/A ^1^	[143]
Methanol extract		*Bacillus mycoides* *Bacillus subtilis* *Staphylococcus aureus* *Enterobacter cloaceae* *Escherichia coli* *Klebsiella pneumoniae* *Aspergillus flavus* *Aspergillus fumigatus* *Botrytis cinerea* *Candida albicans* *Fusarium oxysporum* *Mucor mucedo* *Paecilomyces variotii* *Penicillium purpurescens* *Penicillium verrucosum* *Trichoderma harsianum*	3.12 mg/mL 3.12 mg/mL 3.12 mg/mL 3.12 mg/mL 6.25 mg/mL 6.25 mg/mL 25 mg/mL 12.5 mg/mL 12.5 mg/mL 6.25 mg/mL 25 mg/mL 25 mg/mL 12.5 mg/mL 25 mg/mL 25 mg/mL 25 mg/mL	N/A	
Water extract		*Bacillus mycoides* *Bacillus subtilis* *Staphylococcus aureus* *Enterobacter cloaceae* *Escherichia coli* *Klebsiella pneumoniae* *Aspergillus flavus* *Aspergillus fumigatus* *Botrytis cinerea* *Candida albicans* *Fusarium oxysporum* *Mucor mucedo* *Paecilomyces variotii* *Penicillium purpurescens* *Penicillium verrucosum* *Trichoderma harsianum*	>50 mg/mL against all	N/A	
Fumarprotocetraric acid		*Bacillus mycoides* *Bacillus subtilis* *Staphylococcus aureus* *Enterobacter cloaceae* *Escherichia coli* *Klebsiella pneumoniae* *Aspergillus flavus* *Aspergillus fumigatus* *Botrytis cinerea* *Candida albicans* *Fusarium oxysporum* *Mucor mucedo* *Paecilomyces variotii* *Penicillium purpurescens* *Penicillium verrucosum* *Trichoderma harsianum*	0.062 mg/mL 0.062 mg/mL 0.062 mg/mL 0.062 mg/mL 0.062 mg/mL 0.25 mg/mL 0.25 mg/mL 0.125 mg/mL 0.125 mg/mL 0.25 mg/mL 0.25 mg/mL 0.125 mg/mL 0.25 mg/mL 0.25 mg/mL 0.25 mg/mL 0.25 mg/mL		
Acetone extract	Kopaonik, Serbia	*Bacillus mycoides* *Bacillus subtilis* *Escherichia coli* *Klebsiella pneumoniae* *Staphylococcus aureus* *Aspergillus flavus* *Aspergillus fumigatus* *Candida albicans* *Penicillium purpurescens* *Penicillium verrucosum*	0.78 mg/mL 0.78 mg/mL 1.56 mg/mL 0.78 mg/mL 0.78 mg/mL 25 mg/mL 12.5 mg/mL 6.25 mg/mL 25 mg/mL 25 mg/mL	Atranorin	[138]
Atranorin		*Bacillus mycoides* *Bacillus subtilis* *Escherichia coli* *Klebsiella pneumoniae* *Staphylococcus aureus* *Aspergillus flavus* *Aspergillus fumigatus* *Candida albicans* *Penicillium purpurescens* *Penicillium verrucosum*	0.015 mg/mL 0.0312 mg/mL 1 mg/mL 0.5 mg/mL 0.25 mg/mL 1 mg/mL 0.5 mg/mL 0.25 mg/mL 1 mg/mL 1 mg/mL		
Acetone extract	Serbia	*Bacillus cereus* *Bacillus subtilis* *Staphylococcus aureus* *Escherichia coli* *Proteus mirabilis* *Aspergillus flavus* *Aspergillus niger* *Candida albicans* *Mucor mucedo* *Trichoderma viride* *Cladosporium cladosporioides* *Fusarium oxysporum* *Alternaria alternata* *Penicillium expansum* *Penicillium chrysogenum*	2.5 mg/mL 5 mg/mL 10 mg/mL 20 mg/mL 2.5 mg/mL >40 mg/mL 10 mg/mL 5 mg/mL 10 mg/mL 5 mg/mL 5 mg/mL 10 mg/mL 10 mg/mL 20 mg/mL 10 mg/mL	Hypoprotocetraric acid and fumarprotocetraric acid	[139]
Acetone extract	N/A	*Staphylococcus aureus* *Staphylococcus aureus* *Enterococcus faecalis* *Enterococcus faecium* *Escherichia coli*	250 µg/mL 250 µg/mL ≥250 µg/mL ≥250 µg/mL ≥250 µg/mL	Atranorin and fumarprotocetraric acid	[11]
Acetone extract	Mount Kopaonik, Serbia	*Bacillus mycoides* *Bacillus subtilis* *Staphylococcus aureus* *Enterobacter cloaceae* *Escherichia coli* *Klebsiella pneumoniae* *Aspergillus flavus* *Aspergillus fumigatus* *Botrytis cinerea* *Candida albicans* *Fusarium oxysporum* *Mucor mucedo* *Paecilomyces variotii* *Penicillium purpurescens* *Penicillium verrucosum* *Trichoderma harsianum*	0.78 mg/mL 0.78 mg/mL 0.78 mg/mL 0.78 mg/mL 1.56 mg/mL 0.78 mg/mL 25 mg/mL 12.5 mg/mL 25 mg/mL 6.25 mg/mL 25 mg/mL 25 mg/mL 12.5 mg/mL 25 mg/mL 25 mg/mL 25 mg/mL	N/A	[136]
Methanol extract		*Bacillus mycoides* *Bacillus subtilis* *Staphylococcus aureus* *Enterobacter cloaceae* *Escherichia coli* *Klebsiella pneumoniae* *Aspergillus flavus* *Aspergillus fumigatus* *Botrytis cinerea* *Candida albicans* *Fusarium oxysporum* *Mucor mucedo* *Paecilomyces variotii* *Penicillium purpurescens* *Penicillium verrucosum* *Trichoderma harsianum*	3.12 mg/mL 3.12 mg/mL 3.12 mg/mL 3.12 mg/mL 6.25 mg/mL 6.25 mg/mL 25 mg/mL 12.5 mg/mL 12.5 mg/mL 6.25 mg/mL 25 mg/mL 25 mg/mL 12.5 mg/mL 25 mg/mL 25 mg/mL 25 mg/mL	N/A	
Acetone extract	Mt. Kopaonik, Serbia	*Bacillus mycoides* *Bacillus subtilis* *Staphylococcus aureus* *Enterobacter cloaceae* *Escherichia coli* *Klebsiella pneumoniae* *Aspergillus flavus* *Aspergillus fumigatus* *Botrytis cinerea* *Candida albicans* *Fusarium oxysporum* *Mucor mucedo* *Paecilomyces variotii* *Penicillium purpurescens* *Penicillium verrucosum* *Trichoderma harsianum*	0.78 mg/mL 0.39 mg/mL 0.78 mg/mL 0.78 mg/mL >50 mg/mL 0.78 mg/mL 25 mg/mL 12.5 mg/mL 25 mg/mL 6.25 mg/mL 25 mg/mL >50 mg/mL 12.5 mg/mL 25 mg/mL >50 mg/mL 25 mg/mL	N/A	[134]
Methanol extract		*Bacillus mycoides* *Bacillus subtilis* *Staphylococcus aureus* *Enterobacter cloaceae* *Escherichia coli* *Klebsiella pneumoniae* *Aspergillus flavus* *Aspergillus fumigatus* *Botrytis cinerea* *Candida albicans* *Fusarium oxysporum* *Mucor mucedo* *Paecilomyces variotii* *Penicillium purpurescens* *Penicillium verrucosum* *Trichoderma harsianum*	3.12 mg/mL 3.12 mg/mL 3.12 mg/mL 3.12 mg/mL >50 mg/mL 6.25 mg/mL 25 mg/mL 12.5 mg/mL 12.5 mg/mL 6.25 mg/mL 25 mg/mL >50 mg/mL 12.5 mg/mL 25 mg/mL >50 mg/mL 25 mg/mL		
Acetone extract	Kopaonik, Serbia	*Bacillus mycoides* *Bacillus subtilis* *Staphylococcus aureus* *Enterobacter cloaceae* *Escherichia coli* *Klebsiella pneumoniae* *Aspergillus flavus* *Aspergillus fumigatus* *Botrytis cinerea* *Candida albicans* *Fusarium oxysporum* *Mucor mucedo* *Paecilomyces variotii* *Penicillium purpurescens* *Penicillium verrucosum*	0.78 mg/mL 0.78 mg/mL 0.78 mg/mL 0.78 mg/mL 1.56 mg/mL 0.78 mg/mL 25 mg/mL 12.5 mg/mL 25 mg/mL 6.25 mg/mL 25 mg/mL 25 mg/mL 12.5 mg/mL 25 mg/mL 25 mg/mL	N/A	[144]

^1^ N/A: data not available.

**Table 9:** Antimicrobial activity of extracts of the lichen *Parmotrema perlatum* (Huds) M. Choisy from different localities.

Type of Extract	Harvest Location	Target Microorganism	MIC Values/ZI Diameters	Extract Composition	Reference
Hexane extract	Local market, Noida, Uttar Pradesh, India	*Escherichia coli* *Pseudomonas* sp. *Bacillus subtilis* *Cryptococcus neoformans* *Candida albicans* *Aspergillus niger* *Aspergillus fumigatus*	0.625 mg/mL 1.25 mg/mL 0.312 mg/mL 2.5 mg/mL 2.5 mg/mL 2.5 mg/mL 1.25 mg/mL	N/A ^1^	[145]
Water extract	Giresun, Center, Boztekke Village, Turkey	*Bacillus subtilis* *Bacillus cereus* *Staphylococcus cohnii* *Enterobacter aerogenes* *Proteus vulgaris*	15 mg/mL 15 mg/mL 15 mg/mL 1.875 mg/mL 3.75 mg/mL	N/A	[146]
Ethanol extract		*Bacillus subtilis* *Bacillus cereus* *Gordonia rubripertincta* *Staphylococcus cohnii* *Yersinia pseudotuberculosis* *Saccharomyces cerevisiae* *Candida tropicalis* *Candida albicans*	7.5 mg/mL 7.5 mg/mL 15 mg/mL 7.5 mg/mL 7.5 mg/mL 0.9346 mg/mL 0.46875 mg/mL 0.46875 mg/mL	N/A	
Chloroform extract		*Bacillus subtilis* *Staphylococcus aureus* *Bacillus cereus* *Gordonia rubripertincta* *Staphylococcus cohnii* *Enterobacter aerogenes* *Proteus vulgaris* *Yersinia pseudotuberculosis* *Saccharomyces cerevisiae* *Candida tropicalis* *Candida albicans*	7.5 mg/mL 7.5 mg/mL 3.75 mg/mL 15 mg/mL 7.5 mg/mL 7.5 mg/mL 7.5 mg/mL 7.5 mg/mL 15 mg/mL 0.46875 mg/mL 7.5 mg/mL	N/A	
Hexane extract	Local market of Trivendrum, Kerala, India	*Pseudomonas aeruginosa* *Klebsiella pneumoniae* *Clostridium difficile* *Streptococcus pyogenes*	1310.84 μg/mL 1106.66 μg/mL 238.64 μg/mL 1027.64 μg/mL	D-carvone, Sambucol, Copaene, Cubebene, Viridifloral, Rishitin, Esculetin, Daphnetin.	[149]
Methanol extract		*Pseudomonas aeruginosa* *Klebsiella pneumoniae* *Clostridium difficile* *Streptococcus pyogenes*	185.10 μg/mL 177.84 μg/mL 186.28 μg/mL 202.60 μg/mL	5,7-dihydroxy 4-methyl coumarin, Platambin, Longidione, Gamolenic acid, Stigmastan 3,5-diene.	
Methanol fraction	Shop in Chennai, Tamil Nadu, India	*Lactobacillus plantarum* *Chromobacterium violaceum* *Pseudomonas aeruginosa*	19 ± 0.1 mm 16 ± 0.1 mm 04 ± 0.06 mm	Orcinol (63.26%), and atraric acid (21.38%). Savinin, tetrahydroterotri-L-glutamate, primidolol, ustiloxin, mallotinic acid, and mecambrine	[147]
Chloroform fraction		*Lactobacillus plantarum* *Chromobacterium violaceum* *Pseudomonas aeruginosa*	15 ± 0.05 mm 11 ± 0.05 mm 01 ± 0.05 mm	Benzoic acid (92.47%) and 1,4-ben-zenediol, 2,5-dimethyl (4.518%).	
Hexane fraction		*Lactobacillus plantarum* *Chromobacterium violaceum* *Pseudomonas aeruginosa*	17 ± 0.05 mm 13 ± 0.05 mm 02 ± 0.1 mm	Benzoic acid, 2,4-dihydroxy-3,6-dimethyl-, methyl ester (62.2%), and phosphinous chloride (1.737%).	

^1^ N/A: data not available.

## Data Availability

The original contributions presented in this study are included in the article. Further inquiries can be directed to the corresponding author.

## Abbreviations

The following abbreviations are used in this manuscript:

MICs	Minimum Inhibitory Concentrations
MRSA	Methicillin-Resistant *Staphylococcus aureus*
VOCs	Volatile Organic Compounds
GC-MS	Gas Chromatography–Mass Spectrometry
GC-FID-MS	Gas Chromatography–Flame Ionization Detector–Mass Spectrometry
HPLC	High-Performance Liquid Chromatography
UHPLC-HRMS/MS	Ultra-High-Performance Liquid Chromatography–High-Resolution Tandem Mass Spectrometry
KI	Kovats Retention Index
ZI	Zone of Inhibition
IZD	Inhibition Zone Diameter
RIZD	Relative Inhibition Zone Diameter
DMSO	Dimethyl Sulfoxide
PKS	Polyketide Synthase
ATCC	American Type Culture Collection
t	Trace
N/A	Not Available
